# Regression Equations of Energy Values of Corn, Soybean Meal, and Wheat Bran Developed by Chemical Composition for Growing Pigs

**DOI:** 10.3390/ani10091490

**Published:** 2020-08-24

**Authors:** Pan Yang, Jian Jun Ni, Jin Biao Zhao, Gang Zhang, Cheng Fei Huang

**Affiliations:** 1 State Key Laboratory of Animal Nutrition, College of Animal Science and Technology, China Agricultural University, Beijing 100193, China; ypan23@cau.edu.cn (P.Y.); bs20173040352@cau.edu.cn (J.B.Z.); 18729564631@163.com (G.Z.); 2 China Animal Husbandry Industry Co., Ltd., Beijing 100070, China; Nj_008@163.com; 3 Ministry of Agriculture and Rural Affairs Feed Industry Centre, Beijing 100193, China

**Keywords:** corn, digestible energy, metabolizable energy, prediction equation, soybean meal, wheat bran

## Abstract

**Simple Summary:**

In pig production, feed represents a large part of the total cost, and available energy in feedstuff represents the greatest proportion of this cost. In China, corn, soybean meal (SBM), and wheat bran (WB) are the principal ingredients in diets of pigs because these feedstuffs are widely available in China, have highly available energy, and are generally economical. In practice, the actual digestible energy (DE) and metabolizable energy (ME) of corn, SBM, and WB are unknown. The experimental determination of DE or ME values is expensive, time consuming, and labor intensive. Regression equations to estimate DE and ME in feed ingredients based on chemical composition can be a useful tool in feed ingredient evaluation. Thus, the objectives of this study were to evaluate a wide variety of corn, SBM, and WB to generate the regression equation for DE and ME and to evaluate and compare the energy content of various corn, SBM, and WB samples. A range of regression equations were developed in the present study, and proved adequate to reliably predict the DE and ME of commercially available corn, SBM, and WB for growing pigs. The application of these regression equations is expected to optimize productivity and maximize profits for pork producers.

**Abstract:**

The objectives of this study were to determine the chemical compositions, digestible energy (DE), and metabolizable energy (ME) in corn, soybean meal (SBM) and wheat bran (WB) fed to growing pigs, and to develop regression equations for predicting DE and ME. Three separate experiments were conducted to determine DE and ME of corn, SBM, and WB. The DE and ME in corn were determined directly using 10 barrows allotted to a replicated 5 × 5 Latin square design, and the diets were formulated with one of 10 corn samples. The DE and ME in SBM and WB were determined by difference using two corn basal diets and 10 corn-SBM or 10 corn-SBM-WB diets, which were allotted to a replicated 6 × 6 Latin square design. Ten corn samples were obtained from the main corn producing areas of China. Ten SBM samples were obtained from nine different crushing facilities in nine provinces in China. Ten WB samples were collected from different feed mills of China. Samples were analyzed for dry matter (DM), crude protein (CP), ether extract (EE), ash, neutral detergent fiber (NDF), acid detergent fiber (ADF), gross energy (GE), and soluble carbohydrates (SCHO). The best-fit equations for corn were DE (MJ/kg DM) = 20.18 − 0.76 × EE (%) and ME (MJ/kg DM) = 5.74 + 1.11 × DE (MJ/kg DM) − 0.33 × CP (%) − 0.07 × SCHO (%). The best-fit equations for SBM were DE (MJ/kg DM) = 42.91 − 3.43 × Ash (%) − 0.20 × NDF (%) + 0.09 × ADF (%) and ME (MJ/kg DM) = −21.67 + 0.89 × DE (MJ/kg DM) − 1.06 × GE (MJ/kg DM). The best-fit equations for WB were DE (MJ/kg DM) = −7.09 + 1.54 × CP (%) − 0.25 × NDF (%) − 0.32 × ADF (%) + 0.23 × Ash (%) and ME (MJ/kg DM) = 0.02 + 0.96 × DE (MJ/kg DM). The chemical composition of corn, SBM, and WB can vary substantially from zone to zone, resulting in considerable variation in its available energy value for pig. The DE and ME of corn, SBM and WB for growing pigs can be predicted based on their chemical compositions.

## 1. Introduction

China is the biggest global producer and consumer of pork. The total number of fattening pigs marketed each year is over 650 million, and China consumes and produces more than half of the world’s pig meat (Food and Agriculture Organization Statistic). In large-scale pig production, feed accounts for the largest portion of overall costs, comprising about 70 to 80% of the total costs in pig production. In animal production, energy composition in feed formulation represents the major contribution to the feed cost. Corn, soybean meal (SBM) and wheat bran (WB) are the principal ingredients in diets for pigs [1,2,3,4]. China is one of the largest corn producing countries, almost all of which is consumed domestically. In 2019, China imported more than 80 million tons of soybeans (China Official Report), the SBM is the by–product of the extraction of soybean oil [5]. China is also the leading producer of wheat. The WB is a by–product of wheat processing; more than 20 million tons are produced per year and it is used in large quantities for feed [6]. The main challenge for the pig industry is to maximize feed efficiency while minimizing production costs.

It is essential to estimate the precise energy value of feedstuffs, either for the purposes of minimizing input costs or for adapting the feed supply to the energy requirements of pigs. Although the variability in the available energy of corn, SBM, and WB for pigs is well known [2,3,4,5,6], in application, their actual digestible energy (DE) and metabolizable energy (ME) are unknown. Prediction equations for DE and ME in feed ingredients based on chemical composition can be a useful tool in feed ingredient evaluation [7,8]. We hypothesize that regression equations for the available energy of corn, SBM, and WB samples would allow more precise application than the use of tabular means.

Therefore, the objectives of this study were to (1) determine the DE and ME of corn, SBM, and WB in growing pigs and (2) to develop prediction equations to predict the DE and ME of corn, SBM, and WB based on chemical composition.

## 2. Materials and Methods

All animal handling and care procedures in these studies followed the specifications outlined by the Guide for the Care and Use of Agricultural Animals in Research and Teaching and were approved by the Institutional Animal Care and Use Committee at China Agricultural University (Protocol No. AW13030202–1–1). The experiment was conducted at the Fengning Swine Research Unit of China Agricultural University (Academician Workstation in Chengdejiuyun Agricultural and Livestock Co., Ltd., Chengde, China).

The sources of corn, SBM, and WB are shown in Table 1. To ensure that samples were representative and to increase the variation in the nutrient contents of the corn, SBM and WB used in this study, an investigation of the production and consumption area in China was conducted. After the investigation, ten corn samples were obtained from the main corn producing areas of China. The sources included commercially grown hybrids yielding grain that represented the full range in energy content available from commercially produced hybrids. Ten SBM samples were purchased from nine different crushing facilities in nine provinces in which mainland China’s main SBM-producing regions are located. Ten WB samples were collected from leading commercial Chinese feed mills. These samples were selected to maximize the variation in nutrient composition with the underlying aim of generating good prediction equations that would be useful over a large variety of corn, SBM, and WB samples. Approximately 500 kg per sample were collected for this study. All samples were stored at −20 °C prior to analysis and diet formulation. Before the experiment, subsamples were collected and analyzed for their chemical composition.

### 2.1. Experimental Animal and Diets

Three separate experiments were conducted to determine the DE and ME values of different types of corn, SBM, and WB fed to growing pigs. A total of thirty-four castrated barrows (Duroc × Landrace × Yorkshire; initial body weight of 40.7 ± 2.5 kg) were used for this study and individually housed in stainless steel metabolism crates (1.4 × 0.7 × 0.6 m). For evaluating corn available energy, ten pigs were randomly allotted to a replicated 5 × 5 Latin square design with 5 consecutive periods and each period included 5 d of diet adaptation followed by 5 d of total collection of feces and urine. Ten experimental diets were formulated with one of the 10 different corn types at an inclusion level of 96.0% (Table 2). For evaluating the available energy of SBM, twelve pigs were randomly allotted to a replicated 6 × 6 Latin square design with 6 consecutive periods and each period included 5 d of diet adaptation followed by 5 d of total collection of feces and urine. Experimental diets included a corn basal diet and 10 SBM experimental diets. The SBM test diets were formulated with 24.0% SBM by replacing the corn in the basal diet (Table 2). For evaluating WB available energy, twelve pigs were randomly allotted to a replicated 6 × 6 Latin square design with 6 consecutive periods and each period included 5 d of diet adaptation followed by 5 d of total collection of feces and urine. Experimental diets included a corn-SBM basal diet and 10 WB experimental diets. The WB test diets were formulated with 14.4% WB by replacing corn and SBM in the basal diet (Table 2).

Vitamins and minerals were supplemented in all diets to meet the estimated nutrient requirements for growing pigs [9]. Pigs were provided ad libitum access to water and were fed a daily amount of feed equivalent to 4% of BW determined at the beginning of each period. Daily feed was equally divided into 2 amounts and provided to pigs at 0800 and 1600 h. The amount of feed provided to pigs was recorded during each feeding meal. Feed refusals were weighed after each meal and daily feed consumption was calculated as the difference between provided feed and the feed refusal. The room temperature was maintained at 23 ± 2 °C during all experiments.

### 2.2. Sample Collection and Chemical Analysis Program

All experiments consisted of a 5 d diet adaptation followed by a 5 d period of total collection of feces and urine. During the 5 d collection period, feces were collected into a plastic bag every 4 h from 0800 to 2200 h and then stored at −20 °C. At the end of the experiment, the 5 d of collected feces from each pig were pooled, mixed, and weighed; an 800 g subsample was taken and dried in a forced-draft oven at 65 °C for 72 h. After drying and grinding through a 1 mm screen, fecal subsamples were stored at −20 °C for further chemical analysis. Total urine was collected in plastic buckets attached to funnels located under the metabolism cages at the same time as the fecal collection. Approximately 50 mL of 6 N HCl was added to each bucket to limit microbial growth and reduce the loss of ammonia. Urine volume was recorded daily and a subsample of 10% was collected and stored at −20 °C. At the end of the collection period, urine samples were pooled within each pig and a subsample (about 100 mL) was stored at −20 °C for further analysis.

The chemical analysis of all samples was tested in duplicate. Samples were analyzed for dry matter (DM) (procedure 934.01), crude protein (CP) (procedure 990.03), ether extract (EE; procedure 920.39), and ash (procedure, 942.05) by Association of Official Analytical Chemists (AOAC) methods [10]. Neutral detergent fiber (NDF) and acid detergent fiber (ADF) were determined using fiber bags (Model F57, Ankom Technology, Macedon, NY, USA) and a fiber analyzer (ANKOM200 Fiber Analyzer, Ankom Technology, Macedon, NY, USA) after adaptation of the procedure described by Van Soest et al. [11]. The ADF fraction was analyzed in a separate sample. The gross energy (GE) of corn, SBM and WB, diets, feces, and urine samples were analyzed using an adiabatic oxygen bomb calorimeter (Parr Instruments Co., Moline, IL, USA). Soluble carbohydrates (SCHO) were calculated from analyzed nutrient values as 100 − (protein + fat + ash + NDF) [12,13].

### 2.3. Calculations

The DE and ME content of the 10 corn types were calculated using the direct method as described by Adeola [14]:

DE_d_ = (GE_i_ − GE_f_)/F_i_ (1)

DE_w_ = DE_d_/0.96 (2)

ME_d_ = (GE_i_ − GE_f_ − GE_u_)/F_i_ (3)

ME_w_ = ME_d_/0.96 (4)

where DE_d_ and ME_d_, respectively, are the DE and ME values in each diet (MJ/kg of DM); GE_i_ is the total GE intake of each pig (MJ of DM), and Fi is the actual feed intake (kg) over the 5 d collection period; GE_f_ and GE_u_, respectively, are the total GE content in feces and urine of each pig (MJ of DM) over the 5 d collection period; DE_w_ and ME_w_, respectively, are the DE and ME values in each corn type (MJ of DM) and 0.96 is the percentage of corn in the diet.

The DE and ME of the SBM and WB were also calculated using the difference method of Adeola [14] and Yang et al. [15], according to the following equations:

DE_d_ = (GE_i_ − GE_f_)/F_i_ (5)

DE_dc_ = DE_d_/0.96 (6)

DE_r_ = (DE_d_ − (100% − X%) × DE_dc_)/X% (7)

ME_d_ = (GE_i_ − GE_f_ − GE_u_)/F_i_ (8)

ME_dc_ = ME_d_/0.96 (9)

ME_r_ = (ME_d_ − (100% − X%) × ME_dc_)/X% (10)

where DE_d_ and ME_d_, respectively, are the DE and ME values in each diet (MJ of DM); GE_i_ is the total GE intake of each pig (MJ of DM) calculated as the product of the GE content of the diet over F_i_, which is the actual feed intake over the 5 d collection period; GE_f_ and GE_u_, respectively, are the GE content in feces and urine of each pig (MJ of DM) over the 5 d collection period; DE_dc_ and ME_dc_, respectively, are the adjusted DE and ME in the basal diet (MJ of DM) and 0.96 is the percentage of the ingredients that supplied energy in the diet; DE_r_ and ME_r_, respectively, are the DE and ME values in each soybean meal or wheat bran sample (MJ of DM); and X% is the percentage of energy supplied by SBM or WB in the basal diet.

### 2.4. Statistical Analysis of Data

All of the data were checked for normality and outliers were detected using the UNIVARIATE procedure of SAS (SAS Inst. Inc., Cary, NC, USA). No outliers were found. Correlation coefficients between chemical composition and DE or ME of corn, SBM, and WB were analyzed using the PROC CORR procedure of SAS 9.4 (SAS Inst. Inc., Cary, NC, USA). Prediction equations for swine DE and ME in the corn, SBM, and WB were developed using PROC REG of SAS 9.4 (SAS Inst. Inc., Cary, NC, USA). Statistical differences among the treatments were separated by Tukey’s multiple range test. Treatment means were calculated using the LSMEANS statement. Differences were considered significant if *p* < 0.05. Stepwise regression was used to determine the effect of different chemical constituents on energy values. Variables with *p* ≤ 0.15 were retained in the model. The *R*^2^, Bayesian information criterion (BIC), root mean square error (RMSE) and Akaike’s information criterion (AIC) were used to define the best-fit equations. The equations with the greatest *R*^2^ and the smallest RMSE, and the lower AIC or BIC were chosen as the best-fit models. 

## 3. Results

### 3.1. Chemical Composition in Corn, SBM, and WB

The chemical composition of corns is presented in Table 3. The DM content of the 10 corn samples averaged 86.87% with a range from 86.29 to 87.61%. On a DM basis, the concentrations of CP, ash, EE, and soluble carbohydrates (SCHO) averaged 9.16% (8.07 to 9.69%), 3.24% (1.31 to 6.12%), 4.27% (3.44 to 4.91%), and 70.89% (67.17 to 74.92%), respectively. The variation was particularly high within the main fiber fractions, with a NDF concentration ranging from 11.02 to 14.51% (mean 12.44%), while the values for ADF of corn ranged from 1.75 to 2.52% (mean 2.06%). However, the GE content of the corn samples varied slightly (mean 18.80 MJ/kg, ranging from 18.59 to 19.05 MJ/kg). 

The chemical composition of the 10 lots of soybean meal evaluated showed considerable variation (Table 4). The DM content of the 10 samples averaged 88.77% with a range from 87.52 to 90.14%. On a dry matter basis, the concentrations of CP, ash, EE, NDF, ADF, SCHO, and GE averaged 50.77% (48.80 to 54.67%), 7.08% (6.63 to 7.79%), 1.08% (0.17 to 1.88%), 18.18% (11.70 to 28.29%), 7.38% (6.13 to 10.31%), 22.89% (12.64 to 29.50%) and 19.53 MJ/kg (18.93 to 19.93 MJ/kg), respectively. However, the coefficients of variation of EE, NDF, ADF, and SCHO were higher than those of other ingredients in SBM. 

The chemical composition of WB is present in Table 5. The DM content of the 10 wheat brans averaged 89.22%, with a range from 88.16 to 90.62%. On a dry matter basis, the concentrations of CP, ash, EE, NDF, ADF, SCHO, and GE averaged 20.22% (18.91 to 22.14%), 7.03% (3.91 to 11.17%), 3.04% (2.57 to 4.22%), 39.69% (29.0 to 47.69%), 10.61% (6.93 to 13.0%), 30.02% (19.27 to 45.26%), and 19.2 MJ/kg (18.89 to 19.44 MJ/kg), respectively. Regarding the coefficient of variation in the table, the variation was particularly low for DM, CP, and GE, but higher variation was observed within ash, EE, NDF, ADF, and SCHO of WB.

### 3.2. DE and ME in Corn, SBM, and WB

The digestible and metabolizable energy contents of corn, SBM, and WB are presented in Table 6. There were significant differences (*p* < 0.05) in the DE and ME values in different source of corn, SBM, and WB. The corn DE concentration varied from 16.10 to 17.88 (mean 16.93) MJ/kg DM and the ME concentration varied from 15.57 to 17.49 (mean 16.51) MJ/kg DM. The substantial variation in nutrient composition of SBM led to large variation in the DE and ME content: the DE contents varied from 13.40 (sample 10) to 17.87 (sample 1) MJ/kg DM and the range in ME concentrations varied from 12.75 (sample 4) to 16.95 (sample 5) MJ/ kg DM. In addition, the DE concentration of WB varied from 9.53 to 15.00 (mean 12.23) MJ/kg DM and the ME concentration varied from 9.42 to 14.72 (mean 11.82) MJ/kg DM.

### 3.3. Correlation among Chemical Composition and Energy Values

Correlation coefficients (r) between chemical composition, and the DE and ME content, of the 10 corn samples are shown in Table 7. The SCHO had a negative correlation (*p* < 0.05) with CP and ash. The EE was negatively correlated with DE (*p* < 0.01) and was positively correlated with ADF. The DE and ME of corn were positively correlated (*p* < 0.01). Table 8 shows correlation coefficients between the chemical composition and available energy of SBM; there were positive correlations (r = 0.68, *p* < 0.05) between GE and EE. The SCHO had a negative correlation (r = −0.94, *p* < 0.01) with NDF. Ash was negatively correlated (*p* < 0.05) with DE and ME. The DE and ME of SBM were positively correlated (*p* < 0.01). The correlation coefficients between chemical composition and available energy of WB are presented in Table 9. The DE and ME of WB were positively correlated. There were negative correlations (*p* < 0.01) between ash and DE. The NDF was negatively correlated with DE and ME, and was positively correlated with ash (*p* < 0.05). The SCHO had a positive correlation (*p* < 0.05) with DE and ME, but a negative correlation (*p* < 0.01) with ash and NDF.

### 3.4. Regression Equation

**Figure 1:**
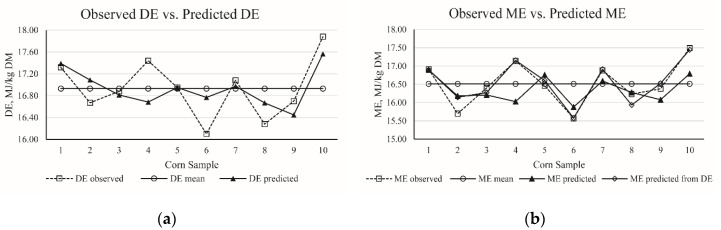
(**a**) The distribution of observed digestible energy (DE) of corn and predicted DE of corn calculated from best-fit equation in present study; (**b**) the distribution of observed metabolism energy of corn (ME), predicted ME of corn from DE, and predicted ME of corn calculated from the best-fit equation in present study.

Based on chemical composition and variables, best regression equations for DE and ME in the corn samples were generated and are presented in Table 10. The *R*^2^ was used as a decision coefficient to avoid over statement could be avoided. The RMSE, AIC, and BIC indicate the equation precision when adding predictors. According to this principal, three equations (No. 1 to 3) for predicting DE values and six equations (No. 4 to 9) for predicting ME values were established. Both DE and ME can be predicted by a single predictor of EE, but with low *R*^2^ values of 0.41 and 0.21, respectively. The *R*^2^ values were improved to 0.43 and 0.27 when the NDF and CP were included in the regression equation of DE and ME, respectively. The highest *R*^2^ of 0.46 was obtained when CP, ash, and EE were included in the regression equation of DE. However, statistical analysis showed that Equations (2) and (3) were not significant. The *R*^2^ was 0.35 in the equation including CP, EE, and GE for predicting ME. When the equation included DE as variable, the ME was accurately predicted (No. 7, 8, and 9). In brief, the best-fit equations for corn were DE (MJ/kg DM) = 20.18 − 0.76 × EE (%) (*R*^2^ = 0.41) and ME (MJ/kg DM) = 5.74 + 1.11 × DE (MJ/kg DM) − 0.33 × CP (%) − 0.07 × SCHO (%) (*R*^2^ = 0.90). Furthermore, the accuracy of the best-fit prediction equations for DE (MJ/kg DM) and ME (MJ/kg DM) in corn samples is presented in Figure 1.

**Figure 2:**
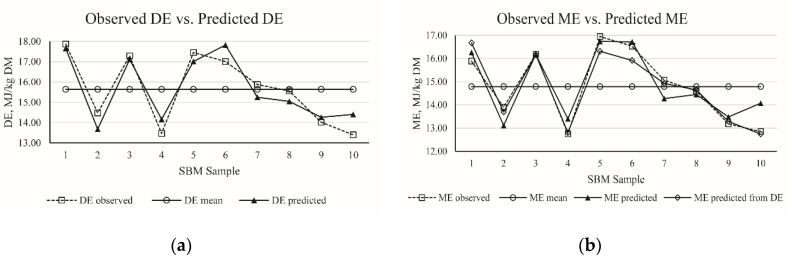
(**a**) The distribution of observed digestible energy (DE) of soybean meal (SBM) and predicted DE of SBM calculated from best-fit equation in present study; (**b**) the distribution of observed metabolism energy (ME) of SBM, predicted ME of SBM from DE, and predicted ME of SBM calculated from the best-fit equation in present study.

Regression equations for SBM of DE and ME were developed by stepwise regression analyses and are listed in Table 11. Considering the statistical criterion of *R*^2^, RMSE, BIC and AIC, five equations (No. 1 to 5) for predicting DE values of SBM and five equations (No. 6 to 10) for predicting ME values of SBM were established. Both DE and ME could be predicted using the single predictor of ash, but with low *R*^2^ values of 0.56 and 0.49, respectively. The *R*^2^ value improved to 0.85 and 0.81 when the NDF was included in the regression equation of DE and ME, respectively. The highest *R*^2^ of 0.86 was obtained when ash, NDF, and ADF were included in the regression equation of DE of SBM. When the equation contained DE as a variable, the ME was accurately predicted (No. 9 and 10). The best-fit equations for SBM were DE (MJ/kg DM) = 42.91 − 3.43 × Ash (%) − 0.20 × NDF (%) + 0.09 × ADF (%) (*R*^2^ = 0.86) and ME (MJ/kg DM) = −21.67 + 0.89 × DE (MJ/kg DM) − 1.06 × GE (MJ/kg DM) (*R*^2^ = 0.97). Furthermore, the accuracy of the best-fit prediction equations for DE (MJ/kg DM) and ME (MJ/kg DM) in corn samples is presented in Figure 2.

**Figure 3:**
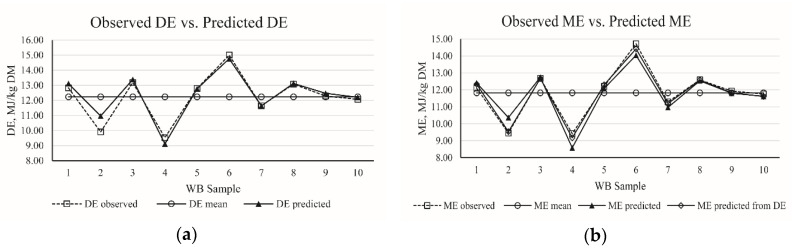
(**a**) The distribution of observed digestible energy (DE) of wheat bran (WB) and predicted DE of WB calculated from best-fit equation in present study; (**b**) the distribution of observed metabolism energy (ME) of WB, predicted ME of WB from DE, and predicted ME of WB calculated from the best-fit equation in present study.

The nine equations generated by the mathematical model for estimating the DE and ME values of WB, based on the *R*^2^, RMSE, BIC, and AIC, are presented in Table 12. Both of DE and ME of WB could be predicted by the single predictor of NDF, and their *R*^2^ values were 0.51 and 0.46, respectively. The *R*^2^ values were improved to 0.79 and 0.74, respectively, when the CP was included in the regression equation of DE and ME. Moreover, the *R*^2^ values of equations for DE and ME could be further improved when the equation was based the three predictors of NDF, CP, and ADF. In addition, if the equation contained DE as a variable, ME could be predicted accurately (No.5). The best-fit equations for WB were DE (MJ/kg DM) = −7.09 + 1.54 × CP (%) − 0.25 × NDF (%) − 0.32 × ADF (%) + 0.23 × Ash (%) (*R*^2^ = 0.86) and ME (MJ/kg DM) = 0.02 + 0.96 × DE (MJ/kg DM) (*R*^2^ = 0.99). Furthermore, the accuracy of best-fit prediction equations for DE (MJ/kg DM) and ME (MJ/kg DM) in corn samples is presented in Figure 3.

## 4. Discussion

### 4.1. Chemical Composition and Energy Variation in Corn, SBM, and WB

In the present study, the average values of chemical composition of corn were in agreement with previous studies [14,16,17], but the variation was particularly high within the main fiber fractions, with the NDF concentration ranging from 11.02 to 14.51%, while the values for ADF of corn ranged from 1.75 to 2.52%. The fiber fractions of corn are variable and dependent on the variety, growing environment, storage duration, and species [18,19,20,21,22]. The feedstuff composition table in the Nutrient Requirement of Swine [9] is probably the most widely and commonly cited reference for ingredient composition used by the scientific community and commercial companies in the formulation of swine diets. The average GE, CP, and EE were comparable to the values from NRC [9], but the average ash and NDF concentrations were slightly greater compared with the NRC [9]. The average concentration of ADF was less than the value from NRC [9]. The CV for CP, EE, ADF, SCHO, and GE were all less than 10%, but greater variations in the content of ash (CV: 53.66%) and NDF (CV: 10.04%) were observed. Some corn types from several provinces had higher content of ash than would normally be expected; this may have resulted from the addition of ground limestone to the meal by processers to improve handling characteristics [2]. The variations in the concentrations of corn can be explained by the fact that the nutrient content of corn growing in different environments is not consistent, as demonstrated in previous research [23]. 

As expected, large differences in the nutrient content of SBM were observed among different origins from which the samples generated. Compared with values from NRC [9], the average GE, ash, EE, and ADF concentrations were comparable, but the average concentrations of ash, NDF, and CP were approximately one percent greater than the related values from NRC [9]. The CV for CP, ash, and GE were within 10%, but wide variations in the content of EE (CV: 45.95%), NDF (CV: 25.88%), ADF (CV: 16.80%), and SCHO (CV: 21.09%) were observed. Generally, the key to optimizing the nutritive values of SBM for feed uses during processing lies in its the areas of dehulling, extraction, and cooking. The variation of EE can be explained by the fact that processing conditions (such as temperature, moisture, and residence time) may result in a large variation of residual oil content [3]. The concentrations of NDF and ADF in some SBM types were very low, which was possibly because soybeans have good rates of hull removal during processing [3].

The WB, which compromises 15% of the wheat grain weight after milling, is a composite multi-layer material made up of several adhesive tissues: outer pericarp, inner pericarp, seed coat, nucellar epidermis, and aleurone layer [4]. Different milling plants have various processes for recovering the different bran layers; the remaining bran tissues from each process result in variation of the wheat bran. As expected, the chemical compositions of WB were variable. Compared with the values from NRC [9], the average GE was comparable. The average CP, ash, and NDF concentrations were slightly higher than the NRC values [9], but the EE and ADF concentrations were slightly less than the NRC values [9]. The CV for DM, CP, and GE were within 10%, but we observed greater variations in the content of ash (CV: 34.40%), EE (CV: 14.31%), NDF (CV: 16.11%), ADF (CV: 17.93%) and SCHO (CV: 28.28%). The variation from the present research can be explained by the fact that processing conditions differed across milling plants.

### 4.2. Available Energy Content of Corn, SBM, and WB

One of the purposes of this study was to increase the variation in the DE and ME contents of the corn types. Large variations in the DE and ME demonstrated that the objective of the current study was successful. The determined DE contents varied by 1.78 MJ/kg or 3.0%, and the ME contents varied by 1.92 MJ/kg or 3.48%. Although the study was designed to ensure access to corn samples with a high degree of variability, the average chemical compositions (DM, ash, EE, and ADF) were in agreement with previous studies [17,21,22,23,24]. The DE of corn in the present study covered the range of published values of 16.35 MJ/kg DM [9], 16.87 MJ/kg DM [25], and 16.45 to 17.49 MJ/kg DM for five Chinese provinces in which corn was produced [17]. The ME value of corn in the present study covered the range of previously published values of 16.08 MJ/kg DM [9], 16.28 MJ/kg DM [25], and 15.89 to 17.12 MJ/kg DM for the five Chinese provinces [17].

In the current study, the substantial variation in nutrient composition of SBM also resulted in large variation in the DE and ME. The DE of soybean meal ranged from 13.4 to 17.87 MJ/kg in the present study, which covers the range of previously published values of 14.57 to 16.68 MJ/kg DM for 22 soybean meals [5] and 15.4 MJ/kg DM [9]. The ME of soybean meal ranged from 12.75 to 16.95 MJ/kg in the present study, which covers the range of previously published values of 14.25 to 16.07 MJ/kg DM for 22 soybean meals [5] and 14.15 MJ/kg DM [9]. The differences in DE and ME values among the 10 classes of SBM were due to differences in their chemical composition (mainly based on EE and fiber content) and the processing methods. Soybean meal with greater oil content (e.g., samples 1, 5, and 6) had greater DE and ME contents than SBM with less oil. The SBM samples from Hunan and Jilin contained greater EE, but the decreased DE and ME values may be due to their higher fiber compositions.

For available energy of WB, the DE concentration varied from 9.53 to 15.00 (mean 12.23) MJ/kg DM and the ME concentration varied from 9.42 to 14.72 (mean 11.82) MJ/kg DM. Compared with previous values reported by Lyu et al. [26], the average DE and ME values from our results were lower, which may be due to the higher dietary fiber content in the current experimental diet, because high fiber content of feed ingredients is responsible for a decline in DE content of feed for pigs [27]. Huang et al. reported that the DE of wheat bran averaged 12.0 MJ/kg DM [6], which was identical with the finding of the present study (12.23 MJ/kg DM). The determined DE content varied by 5.47 MJ/kg or 12.40% and the ME content varied by 5.30 MJ/kg or 12.45%; these values are comparable with the DE and ME values of previously published data. The current results of DE and ME are consistent with the data reported by Hemery et al. [4], in which the DE and ME in wheat bran ranged from 9.74 to 12.11 MJ/kg and 9.49 to 11.72 MJ/kg, respectively, within the 95% confidence intervals obtained for the DE and ME in WB estimated using linear regression. The DE and ME values of wheat bran in the present study were consistent with the previously published values of 9.56 and 10.13 MJ/kg DM, respectively [9].

### 4.3. Correlation among Chemical Characteristics and Energy

In general, dietary lipid (fat) is a critical source of metabolic energy, but correlation coefficients between chemical characteristics and the available energy of corn showed a negative relationship between EE and DE. This may be caused by the small variation in the EE content. The NDF was not significantly correlated with DE or ME content; this was not in accordance with the findings of previous research [17,21], which found that NDF was significantly and negatively correlated with DE in corn. This may be caused by the small variation in the NDF content. Similar to our study, no significant relationship was found between the ADF and DE or ME value [17,21]. Correlation analyses showed that the ME content of corn was highly correlated with DE, which is in line with previous research [27]. 

The NDF and ADF were not significantly correlated with the DE content. This result is not in line with previous research, which found that the NDF, ADF, and CF of SBM were significantly and negatively correlated with DE [5]. This may be caused by the variation in NDF and ADF content. However, similar to our study, no significant relationships were found between the NDF or ADF and the ME value in previous research [5]. The results of several experiments [5,28] indicate that the ME content of SBM was positively correlated with DE, which also was observed in the current experiment.

A linear decrease in DE and ME was observed with the increase in NDF (Figure 1), which is in line with previous studies [6]. For fiber components, the NDF was negatively correlated with the DE and ME content, which corroborated the findings of Noblet and Perez [27] and Huang et al. [6], who reported a negative correlation between the NDF or ADF and the DE or ME values. We found that ash was negatively correlated with the DE value, which is also in agreement with the finding of Huang et al. [6]. Correlation analyses showed that the ME content of WB was positively correlated to its DE.

### 4.4. Regression Equations for DE and ME

Regression equations have been widely used to estimate the DE and ME values of complete diets and individual feedstuffs. The use of such equations potentially decreases the requirement for drawn out and expensive metabolism trials and improves the precision of estimating energy values [27]. For prediction equations of DE and ME values in corn from chemical compositions, multiple and the stepwise regression analysis programs were used. The *R*^2^ was used as a decision coefficient to avoid over statement. The RMSE, BIC, and AIC indicate the equation’s precision when adding predictors. The EE was an important prediction estimator of DE and ME in corn in the present study. This may be because of the significant correlation between EE and the available energy of corn. In the present study, best fit equations were able to predict the change in DE in most samples, with the exception of sample 4. It has been speculated that the size of the starch granule in corn may affect its available energy. Smaller granules of starch have a relatively larger surface area and thus have greater potential for hydrolysis by endogenous amylase. However, the precision of corn DE and ME equation needs to be improved in future studies. Furthermore, it was noted that the best fit equation for corn does not significantly improve upon the results gained from using mean DE. In addition, the results of the present study clearly show that ME content of corn can be accurately predicted from the DE content, because the correlation coefficient between DE and ME was 0.91. Some equations were previously proposed for predicting the ingredients and energy of corn for pigs; however, these involve the determination of a large number of components whose analysis is expensive or time consuming, or demand materials not normally available in the industry, such as starch, calcium, phosphorus, and GE [24]. 

Feed ingredients come from different sources and contain different contents of cellulose, hemicellulose, and lignin, which negatively affect the digestibility of energy. The NDF and ash were two important prediction estimators of DE and ME in SBM. The NDF was considered as the best predictor for DE and ME [27]. Considerable variation of energy content in SBM was observed. Therefore, it was possible to establish the DE and ME regression equations based on the chemical compositions, which can be easily measured and calculated. Under actual conditions, the DE value is available and has a significant positive association with ME. Thus, the ME content of SBM can be accurately predicted from the DE.

The calculated correlation coefficients and regression equations in the current study including chemical composition clearly indicated that the fibrous components predominantly determined the energy values of WB. Because the NDF content was highly and negatively correlated with the DE and ME values of WB, the best single predictor of DE and ME was the NDF. In addition, the inclusion of the NDF content in the equations led to a more precise estimate of DE and ME compared to the inclusion of ADF and CP. Our finding was in agreement with previous studies [6,29,30] comparing different fiber criteria, in which NDF was found to be the most significant factor impacting the DE and ME variation and the key predictor in WB relating to DE and ME values [6,30,31]. Thus, the equation established in the present study used NDF as a prediction estimator in the regression equation for the prediction of DE and ME content. The results of the present study clearly show that ME content of wheat bran can be accurately predicted from the DE content, because the correlation coefficient between DE and ME is 0.99.

## 5. Conclusions

In summary, the concentrations of CP, ash, EE, NDF, ADF, SCHO, DE, and ME in corn, SBM and WB showed considerable differences based on the feeds’ origins. The differences in chemical composition among these feedstuffs, especially the concentrations of fiber, ash, and CP among the feedstuffs, may be responsible for the variability in the nutritive value of corn, SBM, and WB. The best-fit equations for corn were DE (MJ/kg DM) = 20.18 − 0.76 × EE (%) and ME (MJ/kg DM) = 5.74 + 1.11 × DE (MJ/kg DM) − 0.33 × CP (%) − 0.07 × SCHO (%). The best-fit equations for SBM were DE (MJ/kg DM) = 42.91 − 3.43 × Ash (%) − 0.20 × NDF (%) + 0.09 × ADF (%) and ME (MJ/kg DM) = −21.67 + 0.89 × DE (MJ/kg DM) − 1.06 × GE (MJ/kg DM). The best-fit equations for WB were DE (MJ/kg DM) = −7.09 + 1.54 × CP (%) − 0.25 × NDF (%) − 0.32 × ADF (%) + 0.23 × Ash (%) and ME (MJ/kg DM) = 0.02 + 0.96 × DE (MJ/kg DM). We suggest that the chemical compositions of corn, SBM, and WB of different origins should be taken into consideration when formulating feed for swine. Research to confirm the accuracy of these equations is needed in the future.

## Figures and Tables

**Table 1:** Origin of corn, soybean meal (SBM), and wheat bran (WB) used in the study.

Corn No.	Source	SBM No.	Source	WB No.	Source
1	Gansu	1	Gansu	1	Gansu
2	Hebei	2	Hebei	2	Hebei
3	Heilongjiang	3	Heilongjiang	3	Heilongjiang
4	Henan	4	Henan	4	Henan 1
5	Hubei	5	Hubei 1	5	Henan 2
6	Hunan	6	Hubei 2	6	Hubei
7	Jilin	7	Hunan	7	Hunan
8	Liaoning	8	Jilin	8	Jilin
9	Shandong	9	Shandong	9	Shandong
10	Xinjiang	10	Xinjiang	10	Xinjiang

**Table 2:** Ingredient composition of the experimental (Exp.) diets (% as fed basis).

Ingredients	Exp. 1	Exp. 2		Exp. 3	
	Corn Diet	Basal Diet	SBM Diet	Basal Diet	WB Diet
Corn	96.0	96.0	72.0	77.4	65.8
Soybean meal (SBM)	–	–	24.0	18.6	15.8
Wheat bran (WB)	–	–	–	–	14.4
Dicalcium phosphate	1.8	1.8	1.8	1.8	1.8
Limestone	0.9	0.9	0.9	0.9	0.9
Salt	0.3	0.3	0.3	0.3	0.3
Premix ^1^	1.0	1.0	1.0	1.0	1.0
Total	100.0	100.0	100.0	100.0	100.0

^1^ Premix provided the following per kg of complete diet for growing pigs: vitamin A, 5000 IU; vitamin D_3_, 1350 IU; vitamin E, 13.5 IU; vitamin K_3_, 1.45 mg; vitamin B_12_, 9 μg; riboflavin, 2.7 mg; pantothenic acid, 8.0 mg; niacin, 16 mg; choline chloride, 280 mg; folacin, 0.3 mg; thiamine 0.7 mg; pyridoxine 1.35 mg; biotin, 20 μg; Mn, 15.0 mg (MnO); Fe, 70 mg (FeSO_4_·H_2_O); Zn, 65 mg (ZnO); Cu, 25 mg (CuSO_4_·5H_2_O); I, 0.3 mg (KI); Se, 0.2 mg (Na_2_SeO_3_).

**Table 3:** Analyzed chemical composition of corn (%, DM basis) ^1^.

No. ^2^	GE (MJ/kg)	Chemical Composition						
		DM	CP	Ash	EE	NDF	ADF	SCHO ^3^
1	18.70	87.16	8.56	1.31	3.67	11.54	1.86	74.92
2	18.73	87.61	9.69	5.12	4.07	11.11	1.88	70.00
3	18.80	87.34	9.29	1.31	4.43	11.55	2.52	73.43
4	18.85	87.05	9.60	5.45	4.60	12.10	2.07	68.26
5	19.01	86.89	8.95	1.68	4.25	13.74	2.14	71.38
6	18.64	87.34	9.46	1.37	4.49	14.51	2.22	70.17
7	18.59	86.29	8.07	3.50	4.22	11.02	2.04	73.20
8	19.05	86.29	9.62	6.12	4.62	12.47	2.00	67.17
9	18.84	86.42	8.97	3.25	4.91	14.42	2.16	68.46
10	18.78	86.33	9.42	3.25	3.44	11.97	1.75	71.92
Mean	18.80	86.87	9.16	3.24	4.27	12.44	2.06	70.89
Min.	18.59	86.29	8.07	1.31	3.44	11.02	1.75	67.17
Max.	19.05	87.61	9.69	6.12	4.91	14.51	2.52	74.92
SD ^2^	0.14	0.48	0.50	1.74	0.42	1.25	0.21	2.39
CV ^3^	0.75	0.55	5.45	53.66	9.93	10.04	9.99	3.38

^1^ DM = dry matter; CP = crude protein; EE = ether extract; NDF = neutral detergent fiber; ADF = acid detergent fiber; GE = gross energy; SCHO = soluble carbohydrates; Min. = minimum; Max. = Maximum; SD = standard deviation; CV = coefficient of variation. ^2^ Origins of corn are described in Table 1. ^3^ Calculated from analyzed nutrient values as 100 — (protein + EE + ash + NDF).

**Table 4:** Analyzed chemical composition of soybean meal (%, DM basis) ^1^.

No. ^2^	GE (MJ/kg)	Chemical Composition						
		DM	CP	Ash	EE	NDF	ADF	SCHO ^3^
1	19.93	89.14	48.80	6.65	1.88	15.04	6.27	27.62
2	19.53	87.52	51.41	7.79	0.88	15.95	7.53	23.97
3	19.54	88.12	50.06	6.63	0.91	18.46	7.04	23.94
4	19.48	89.27	49.71	7.46	0.90	20.44	10.31	21.49
5	18.93	88.97	51.16	7.05	0.58	11.70	6.93	29.50
6	19.58	89.15	49.95	6.75	1.67	12.87	7.10	28.76
7	19.69	89.18	50.89	6.65	1.54	28.29	9.00	12.64
8	19.46	88.19	50.50	7.18	1.27	19.38	7.10	21.67
9	19.76	87.97	54.67	7.55	0.95	16.51	6.13	20.33
10	19.36	90.14	50.54	7.13	0.17	23.15	6.43	19.00
Mean	19.53	88.77	50.77	7.08	1.08	18.18	7.38	22.89
Min.	18.93	87.52	48.80	6.63	0.17	11.70	6.13	12.64
Max.	19.93	90.14	54.67	7.79	1.88	28.29	10.31	29.50
SD ^2^	0.25	0.75	1.49	0.40	0.49	4.71	1.24	4.83
CV ^3^	1.30	0.84	2.93	5.58	45.95	25.88	16.80	21.09

^1^ DM = dry matter; CP = crude protein; EE = ether extract; NDF = neutral detergent fiber; ADF = acid detergent fiber; GE = gross energy; SCHO = soluble carbohydrates; Min. = minimum; Max. = Maximum; SD = standard deviation; CV = coefficient of variation. ^2^ Origins of corn are described in Table 1. ^3^ Calculated from analyzed nutrient values as 100 − (protein + EE + ash + NDF).

**Table 5:** Analyzed chemical composition of wheat bran (%, DM basis) ^1^.

No. ^2^	GE (MJ/kg)	Chemical Composition						
		DM	CP	Ash	EE	NDF	ADF	SCHO ^3^
1	19.44	88.52	18.91	4.01	2.82	29.00	8.10	45.26
2	19.19	88.62	20.55	8.51	2.93	46.13	12.55	21.88
3	19.24	89.02	20.24	5.79	2.71	33.08	11.72	38.18
4	19.28	89.95	19.42	10.34	3.28	47.69	13.00	19.27
5	19.42	88.16	22.14	5.44	2.84	46.40	12.14	23.17
6	19.12	90.13	21.15	3.91	2.57	32.89	10.61	39.49
7	18.94	89.64	19.68	5.80	2.92	40.06	9.08	31.54
8	18.89	88.69	19.98	8.86	3.09	35.55	11.58	32.52
9	19.43	88.87	20.45	6.44	4.22	40.40	10.37	28.50
10	19.01	90.62	19.72	11.17	3.02	45.71	6.93	20.38
Mean	19.20	89.22	20.22	7.03	3.04	39.69	10.61	30.02
Min.	18.89	88.16	18.91	3.91	2.57	29.00	6.93	19.27
Max.	19.44	90.62	22.14	11.17	4.22	47.69	13.00	45.26
SD ^2^	0.19	0.77	0.88	2.42	0.44	6.39	1.90	8.49
CV ^3^	1.01	0.86	4.33	34.40	14.31	16.11	17.93	28.28

^1^ DM = dry matter; CP = crude protein; EE = ether extract; NDF = neutral detergent fiber; ADF = acid detergent fiber; GE = gross energy; SCHO = soluble carbohydrates; Min. = minimum; Max. = Maximum; SD = standard deviation; CV = coefficient of variation. ^2^ Origins of corn are described in Table 1. ^3^ Calculated from analyzed nutrient values as 100 — (protein + EE + ash + NDF).

**Table 6:** Energy values for corn, soybean meal and wheat bran (MJ/kg, Dry matter basis) ^1^.

No.	Corn		Soybean Meal		Wheat Bran	
	DE	ME	DE	ME	DE	ME
1	17.32 ^a,b^	16.91 ^a,b^	17.87 ^a^	15.89 ^b,c^	12.83 ^b,c^	12.11 ^b,c,d^
2	16.67 ^b,c,d^	15.70 ^c^	14.48 ^c^	13.90 ^e,f^	9.91 ^e^	9.45 ^e^
3	16.88 ^b,c,d^	16.40 ^b,c^	17.29 ^a^	16.19 ^a,b^	13.18 ^b^	12.68 ^b^
4	17.44 ^a,b^	17.14 ^a,b^	13.47 ^d^	12.75 ^g^	9.53 ^e^	9.42 ^e^
5	16.95 ^b,c^	16.46 ^b,c^	17.46 ^a^	16.95 ^a^	12.79 ^b,c^	12.21 ^b,c^
6	16.10 ^d^	15.57 ^c^	17.01 ^a^	16.53 ^a,b^	15.00 ^a^	14.72 ^a^
7	17.08 ^a,b,c^	16.88 ^a,b^	15.88 ^b^	15.06 ^c,d^	11.64 ^d^	11.29 ^d^
8	16.28 ^c,d^	16.23 ^b,c^	15.56 ^b^	14.60 ^d,e^	13.08 ^b^	12.59 ^b,c^
9	16.70 ^b,c,d^	16.38 ^b,c^	14.02 ^c,d^	13.18 ^f,g^	12.29 ^c,d^	11.93 ^b,c,d^
10	17.88 ^a^	17.49 ^a^	13.40 ^d^	12.85 ^g^	12.07 ^c,d^	11.76 ^c,d^
Mean	16.93	16.51	15.64	14.79	12.23	11.82
Min.	16.10	15.57	13.40	12.75	9.53	9.42
Max.	17.88	17.49	17.87	16.95	15.00	14.72
SD	0.51	0.57	1.63	1.49	1.52	1.47
CV	0.03	0.03	0.10	0.10	0.12	0.12
SEM	0.16	0.19	0.18	0.18	0.16	0.17
*p*-value	<0.01	<0.01	<0.01	<0.01	<0.01	<0.01

^1 a,b,c,d,e,f,g^ Values in a column without a common superscript are different, *p* < 0.05. DE = digestible energy; ME = metabolizable energy.

**Table 7:** Correlation coefficients among chemical compositions and energy in the corn ^1^.

Items	DE	ME	CP	Ash	EE	NDF	ADF	GE	SCHO
DE	1.00								
ME	0.92 **	1.00							
CP	−0.23	−0.32	1.00						
Ash	−0.04	0.04	0.43	1.00					
EE	−0.64 *	−0.45	0.17	0.25	1.00				
NDF	−0.47	−0.37	0.16	−0.27	0.51	1.00			
ADF	−0.44	−0.37	0.01	−0.40	0.65 *	0.31	1.00		
GE	−0.11	0.02	0.41	0.33	0.32	0.29	0.08	1.00	
SCHO	0.36	0.26	−0.67 *	−0.75 **	−0.54	−0.40	0.14	−0.53	1.00

^1^ DE = digestible energy; ME = metabolizable energy; CP = crude protein; EE = ether extract; NDF = neutral detergent fiber; ADF = acid detergent fiber; GE = gross energy; SCHO = soluble carbohydrates; *, ** represent *p* < 0.05 and *p* < 0.01, respectively.

**Table 8:** Correlation coefficients among chemical compositions and energy in the soybean meal ^1^.

Items	DE	ME	CP	Ash	EE	NDF	ADF	GE	SCHO
DE	1.00								
ME	0.97 **	1.00							
CP	−0.42	−0.36	1.00						
Ash	−0.75 **	−0.70 *	0.55	1.00					
EE	0.54	0.43	−0.32	−0.48	1.00				
NDF	−0.44	−0.48	−0.04	−0.12	−0.08	1.00			
ADF	−0.31	−0.27	−0.27	0.12	0.06	0.47	1.00		
GE	0.01	−0.17	−0.01	−0.17	0.68 *	0.22	−0.06	1.00	
SCHO	0.57	0.59	−0.28	−0.08	0.11	−0.94 **	−0.39	−0.26	1.00

^1^ DE = digestible energy; ME = metabolizable energy; CP = crude protein; EE = ether extract; NDF = neutral detergent fiber; ADF = acid detergent fiber; GE = gross energy; SCHO = soluble carbohydrates; *, ** represent *p* < 0.05 and *p* < 0.01, respectively.

**Table 9:** Correlation coefficients among chemical compositions and energy in the wheat bran ^1^.

Items	DE	ME	CP	Ash	EE	NDF	ADF	GE	SCHO
DE	1.00								
ME	0.99 **	1.00							
CP	0.34	0.34	1.00						
Ash	−0.64 **	−0.60	−0.29	1.00					
EE	−0.31	−0.29	−0.11	0.28	1.00				
NDF	−0.71 *	−0.68 *	0.25	0.67 *	0.29	1.00			
ADF	−0.29	−0.28	0.44	0.07	0.03	0.28	1.00		
GE	−0.06	−0.10	0.20	−0.38	0.28	0.00	0.19	1.00	
SCHO	0.70 *	0.66 *	−0.20	−0.77 **	−0.34	−0.98 **	−0.28	0.08	1.00

^1^ DE = digestible energy; ME = metabolizable energy; CP = crude protein; EE = ether extract; NDF = neutral detergent fiber; ADF = acid detergent fiber; GE = gross energy; SCHO = soluble carbohydrates; *, ** represent *p* < 0.05 and *p* < 0.01, respectively.

**Table 10:** Regression equations for digestible energy (DE) and metabolizable energy (ME) in corn from and chemical characteristics ^1^.

No.	Regression Equations	Model Statistics ^2^				
		*R* ^2^	AIC	BIC	RMSE	*p*-Value
1	DE (MJ/kg DM) = 20.18 − 0.76 × EE (%)	0.41	−14.81	−19.76	0.44	0.05
2	DE (MJ/kg DM) = 20.65 − 0.64 × EE (%) − 0.08 × NDF (%)	0.43	−13.27	−13.32	0.46	0.14
3	DE (MJ/kg DM) = 21.97 − 0.21 × CP (%) + 0.06 × Ash (%) − 0.78 × EE (%)	0.46	−11.68	−6.13	0.48	0.27
4	ME (MJ/kg DM) = 19.13 − 0.61 × EE (%)	0.21	−9.37	−12.89	0.57	0.19
5	ME (MJ/kg DM) = 21.55 − 0.29 × CP (%) − 0.55 × EE (%)	0.27	−8.19	−13.54	0.59	0.34
6	ME (MJ/kg DM) = −2.21 − 0.43 × CP (%) − 0.67 × EE (%) + 1.35 × GE (MJ/kg DM)	0.35	−7.41	−14.52	0.60	0.42
7	ME (MJ/kg DM) = 1.07 − 1.03 × DE (MJ/kg DM)	0.84	−25.38	−27.15	0.26	<0.01
8	ME (MJ/kg DM) = 0.75 + 1.01 × DE (MJ/kg DM) − 0.14 × CP (%)	0.85	−24.29	−27.21	0.26	<0.01
9	ME (MJ/kg DM) = 5.74 + 1.11 × DE (MJ/kg DM) − 0.33 × CP (%) − 0.07 × SCHO (%)	0.90	−25.84	−28.64	0.24	<0.01

^1^ Regression equations were developed by stepwise regression analyses. ^2^ AIC = Akaike’s information criterion; BIC = Bayesian information criterion; RMSE = root mean square error; DM = dry matter; CP = crude protein; EE = ether extract; NDF = neutral detergent fiber; ADF = acid detergent fiber; GE = gross energy; SCHO = soluble carbohydrates.

**Table 11:** Regression equations for digestible energy (DE) and metabolizable energy (ME) in soybean meal from and chemical characteristics ^1^.

No.	Regression Equations	Model Statistics ^2^				
		*R* ^2^	AIC	BIC	RMSE	*p*-Value
1	DE (MJ/kg DM) = 37.57 − 3.10 × Ash (%)	0.56	5.53	8.16	1.21	0.01
2	DE (MJ/kg DM) = 32.44 − 2.93 × Ash (%) − 0.17 × SCHO (%)	0.82	−1.40	4.84	0.83	<0.01
3	DE (MJ/kg DM) = 42.99 − 3.38 × Ash (%) − 0.19 × NDF (%)	0.85	−3.49	0.90	0.74	<0.01
4	DE (MJ/kg DM) = 42.59 + 0.01 × CP (%) − 3.40 × Ash (%) + 0.19 × NDF (%)	0.85	−1.50	7.94	0.80	<0.01
5	DE (MJ/kg DM) = 42.91 − 3.43 × Ash (%) − 0.20 × NDF (%) + 0.09 × ADF (%)	0.86	−1.72	7.54	0.79	<0.01
6	ME (MJ/kg DM) = 33.53 − 2.65 × Ash (%)	0.49	5.25	3.08	1.19	0.02
7	ME (MJ/kg DM) = 38.73 − 2.91 × Ash (%) − 0.18 × NDF (%)	0.81	−2.75	−3.01	0.77	<0.01
8	ME (MJ/kg DM) = 61.22 − 3.02 × Ash (%) − 0.17 × NDF (%) − 1.12 GE (MJ/kg DM)	0.85	−2.73	−2.36	0.75	<0.01
9	ME (MJ/kg DM) = 0.95 + 0.88 × DE (MJ/kg DM)	0.94	−15.45	−11.46	0.42	<0.01

^1^ Regression equations were developed by stepwise regression analyses. ^2^ AIC = Akaike’s information criterion; BIC = Bayesian information criterion; RMSE = root mean square error; DM = dry matter; CP = crude protein; EE = ether extract; NDF = neutral detergent fiber; ADF = acid detergent fiber; GE = gross energy; SCHO = soluble carbohydrates.

**Table 12:** Regression equations for digestible energy (DE) and metabolizable energy (ME) in wheat bran from and chemical characteristics ^1^.

No.	Regression Equations	Model Statistics ^2^				
		*R* ^2^	AIC	BIC	RMSE	*p*-Value
1	DE (MJ/kg DM) = 18.93 − 0.17 × NDF (%)	0.51	5.30	5.32	1.19	0.02
2	DE (MJ/kg DM) = 0.90 + 0.96 × CP (%) − 0.20 × NDF (%)	0.79	−1.30	2.75	0.83	<0.01
3	DE (MJ/kg DM) = −1.75 + 1.21 × CP (%) − 0.19 × NDF (%) − 0.30 × ADF (%)	0.90	−6.50	3.46	0.63	<0.01
4	DE (MJ/kg DM) = −7.09 + 1.54 × CP (%) − 0.25 × NDF (%) − 0.32 × ADF (%) + 0.23 × Ash (%)	0.94	−9.90	0.62	0.52	<0.01
5	ME (MJ/kg DM) = 0.02 + 0.96 × DE (MJ/kg DM)	0.99	−33.48	−35.74	0.17	<0.01
6	ME (MJ/kg DM) = 18.03 − 0.16 × NDF (%)	0.46	5.51	1.54	1.21	0.03
7	ME (MJ/kg DM) = 0.74 + 0.92 × CP (%) − 0.19 × NDF (%)	0.74	0.17	−5.73	0.89	<0.01
8	ME (MJ/kg DM) = −1.81 + 1.16 × CP (%) − 0.17 × NDF (%) − 0.29 × ADF (%)	0.85	−3.11	−10.91	0.74	<0.01
9	ME (MJ/kg DM) = −7.57 + 1.52 × CP (%) − 0.25 × NDF (%) − 0.31 × ADF (%) + 0.25 × Ash (%)	0.90	−5.33	−14.98	0.66	<0.01

^1^ Regression equations were developed by stepwise regression analyses. ^2^ AIC = Akaike’s information criterion; BIC = Bayesian information criterion; RMSE = root mean square error; DM = dry matter; CP = crude protein; EE = ether extract; NDF = neutral detergent fiber; ADF = acid detergent fiber; GE = gross energy; SCHO = soluble carbohydrates.
